# The Rhizobial Microbiome from the Tropical Savannah Zones in Northern Côte d’Ivoire

**DOI:** 10.3390/microorganisms9091842

**Published:** 2021-08-30

**Authors:** Sara Laetitia Elphège Gnangui, Romain Kouakou Fossou, Anicet Ebou, Chiguié Estelle Raïssa Amon, Dominique Kadio Koua, Claude Ghislaine Zaka Kouadjo, Don A. Cowan, Adolphe Zézé

**Affiliations:** 1 Laboratoire de Biotechnologies Végétale et Microbienne (LBVM), Unité Mixte de Recherche et d’Innovation en Sciences Agronomiques et Génie Rural, Institut National Polytechnique Felix Houphouët-Boigny, Yamoussoukro 1093, Côte d’Ivoire; laetitiasara1987@gmail.com (S.L.E.G.); anicet.ebou@gmail.com (A.E.); emmamon9@gmail.com (C.E.R.A.); youhedeba@gmail.com (A.Z.); 2 Équipe Bioinformatique, Département de Formation et de Recherche Agriculture et Ressources Animales, Institut National Polytechnique Félix Houphouët-Boigny, Yamoussoukro 1313, Côte d’Ivoire; dominique.koua@inphb.ci; 3 Laboratoire Central de Biotechnologies, Centre National de la Recherche Agronomique, 01 Abidjan 1740, Côte d’Ivoire; claudghis@gmail.com; 4 Centre for Microbial Ecology and Genomics, Department of Biochemistry, Genetics and Microbiology, University of Pretoria, Pretoria 0002, South Africa; don.cowan@up.ac.za

**Keywords:** african soil microbiome (AfSM) project, savannah, *Bradyrhizobium*, high-throughput amplicon sequencing (HTAS), 16S rDNA variable regions, V5-V7 region

## Abstract

Over the past decade, many projects have been initiated worldwide to decipher the composition and function of the soil microbiome, including the African Soil Microbiome (AfSM) project that aims at providing new insights into the presence and distribution of key groups of soil bacteria from across the African continent. In this national study, carried out under the auspices of the AfSM project, we assessed the taxonomy, diversity and distribution of rhizobial genera in soils from the tropical savannah zones in Northern Côte d’Ivoire. Genomic DNA extracted from seven sampled soils was analyzed by sequencing the V4-V5 variable region of the 16S rDNA using Illumina’s MiSeq platform. Subsequent bioinformatic and phylogenetic analyses showed that these soils harbored 12 out of 18 genera of Proteobacteria harboring rhizobia species validly published to date and revealed for the first time that the *Bradyrhizobium* genus dominates in tropical savannah soils, together with *Microvirga* and *Paraburkholderia*. In silico comparisons of different 16S rRNA gene variable regions suggested that the V5-V7 region could be suitable for differentiating rhizobia at the genus level, possibly replacing the use of the V4-V5 region. These data could serve as indicators for future rhizobial microbiome explorations and for land-use decision-making.

## 1. Introduction

Since the advent of sequencing technologies, the determination of microbial diversity has become a major topic of interest [1]. Over the last decade, for example, many small- or broad- scales initiatives have been launched around the world to decipher the composition and function of soil microbiome [2,3,4,5,6,7], including the African Soil Microbiome (AfSM) Project implemented in Sub-Saharan Africa (SSA) [8,9]. This unique multi-national project, implemented in a dozen SSA countries, is the first such study to ever be undertaken in Africa at this scale [9,10]. It was launched in 2016 to provide new insights into the presence and the distribution of key groups of soil bacteria, including the rhizobia, by using the high-throughput amplicon sequencing (HTAS) and phylogeny of the 16S rRNA gene [9,10].

Rhizobia are Gram-negative saprophytic *Alpha-* and *Beta-proteobacteria* that play a key role in nitrogen biochemical cycling [11,12]. They form a polyphyletic group of bacteria among the lineages of prokaryotes capable of reducing atmospheric dinitrogen (N_2_) into ammonia (NH_3_) using their nitrogenase enzyme complex [13,14,15]. The symbiotic reduction of N_2_ by diazotrophic rhizobia happened in microoxic conditions of the mature nodule during endosymbiosis with legumes (exceptionally, with the non-legume *Parasponia* species) [16,17,18]. During the nodulation process, host plants actively screen infecting bacteria for non-compatible or hitchhiking strains via the exchange of multiple molecular signals [16,17,18]. In spite of such selectivity in the recruitment of symbionts, rhizobia isolated from nodules belong to remarkably diverse microbial taxa [15,19]. They are scattered across 18 genera in the families: *Brucellaceae* (genus *Ochrobactrum*), *Devosiaceae* (formely *Hyphomicrobiaceae:* [20,21]) (*Devosia*), *Methylobacteriaceae* (*Methylobacterium*, *Microvirga*), *Nitrobacteraceae* (formely *Bradyrhizobiaceae*: [21,22]) (*Bradyrhizobium*), *Phyllobacteriaceae* (*Aminobacter*, *Mesorhizobium*, *Phyllobacterium*), *Rhizobiaceae* [*Allorhizobium*, *Ensifer* (syn. *Sinorhizobium*), *Neorhizobium*, *Pararhizobium*, *Rhizobium*, *Shinella*], *Xanthobacteraceae* (*Azorhizobium*) and *Burkholderiaceae* (*Cupriavidus*, *Paraburkholderia*, *Trinickia*); the last three genera belonging to the class of *Betaproteobacteria* [15]. *Bradyrhizobium* [23] is considered to be the largest group of rhizobia in terms of frequency of isolation [19] and likely to be the ancestor of all rhizobia [24,25,26], while *Rhizobium* [27] has the highest number of described species ([28], https://lpsn.dsmz.de/genus/rhizobium; accessed on 19 February 2021). In Sub-Saharan Africa (SSA), the dominance of culturable *Bradyrhizobium* strains has been repeatedly reported [29,30], including in Côte d’Ivoire [31,32,33]. However, most of these studies focused on microsymbionts of legume species known to be natural hosts for *Bradyrhizobium* (e.g., *Cajanus cajan, Glycine max, Macroptilium atropurpureum* and *Vigna unguiculata*) [30,32,34]. A pioneering study of soil microbiomes from African savannah woodlands carried out in Mozambique, based on both culture-dependent (isolation directly from soil or from *V. unguiculata* used as trapping host) and culture-independent (HTAS analysis of the 16S rDNA’s V3-V4 region from soil DNA) methods, revealed the presence and dominance of the *Bradyrhizobium* genus with a trapping assay only [35]. Similar results were recently obtained from savannah soils in Botswana [36]. The apparent paucity of the cosmopolitan *Bradyrhizobium* genus in these recent HTAS analyses and the limited distribution pattern on the African continent of *Burkholderia* *sensu lato* (*s.l.*) from biogeographical surveys [37] raises a number of questions, including the global distribution pattern of the rhizobia population in the Sub-Saharan Africa soils [37]. There are also concerns about the discriminatory power of some variable (V) regions and corresponding primers to accurately estimate the relative abundance of some genera of N-cycling bacteria from environmental samples, as indicated elsewhere [38]. It appears, therefore, that conclusions of the dominance of *Bradyrhizobium* strains in soils must be treated with caution, and further investigation is required. For example, recent studies suggest the emergence of beta-rhizobia, such as *Paraburkholderia* species, as potential nodulators of various indigenous legumes, including many species traditionally reported to be preferentially nodulated by *Bradyrhizobium* (e.g., the South African *Acacia karroo* and *Aspalathus linearis*) [39,40].

The identification of rhizobia from soils using the high-throughput amplicon sequencing and phylogeny of the 16S rRNA gene was found not sufficient to confirm their ability to nodulate and/or to fix nitrogen [41,42]. However, data on their presence and distribution in soils are valuable for creating a baseline for further studies [8] and may provide new information for land use and crop management decision-making [10,43,44]. Thus, the HTAS of rhizobial communities has been assessed in several ecosystems worldwide, such as temperate arable soils in Poland [45] and coniferous forest soils in North America [41]. However, few HTAS-based studies have been carried out in Africa, and little is known about the global composition and distribution of the rhizobial communities inhabiting soils in African tropical zones, including savannah soils.

In Côte d’Ivoire, the savannah biome covers approximately 54% of the total area of the country [46]. The vegetation of this region is diversified and varies from woodlands to grasslands and occasional patches of dry scrub in the far north [47]. Narrow gallery forests extend along watercourses and drainage lines. All these vegetation types are traditionally divided into three zones, namely the Sudan savannah in the far north, the Sub-Sudan savannah in the north, both of which constitute more than two-thirds of the entire savannah region, and the Guinean savannah. The Guinean savannah, which is located in the southern part of the savannah biome, is sometimes referred to as the transition zone, even though the entire savannah region is transitional between the narrow belt of forest paralleling the coastline and the Sahara [47]. While the Guinean savannah zone has been well studied, including the phylogenetic relationships, ecological niches and functional roles of *N*-cycling bacteria [48,49], the biodiversity and ecology of microorganisms from the two other savannah zones remain relatively unexplored.

The aim of this study was to assess the taxonomy, diversity and distribution of rhizobial genera in soils from the Sudan and the Sub-Sudan tropical savannah zones in Northern Côte d’Ivoire using high-throughput sequencing of the 16S rDNA variable V4-V5 region. In addition, the different 16S rRNA gene variable regions were compared in silico to assess their effectiveness for differentiating all the genera of rhizobia validly published to date.

## 2. Materials and Methods

### 2.1. The Study Area

**Figure 1:**
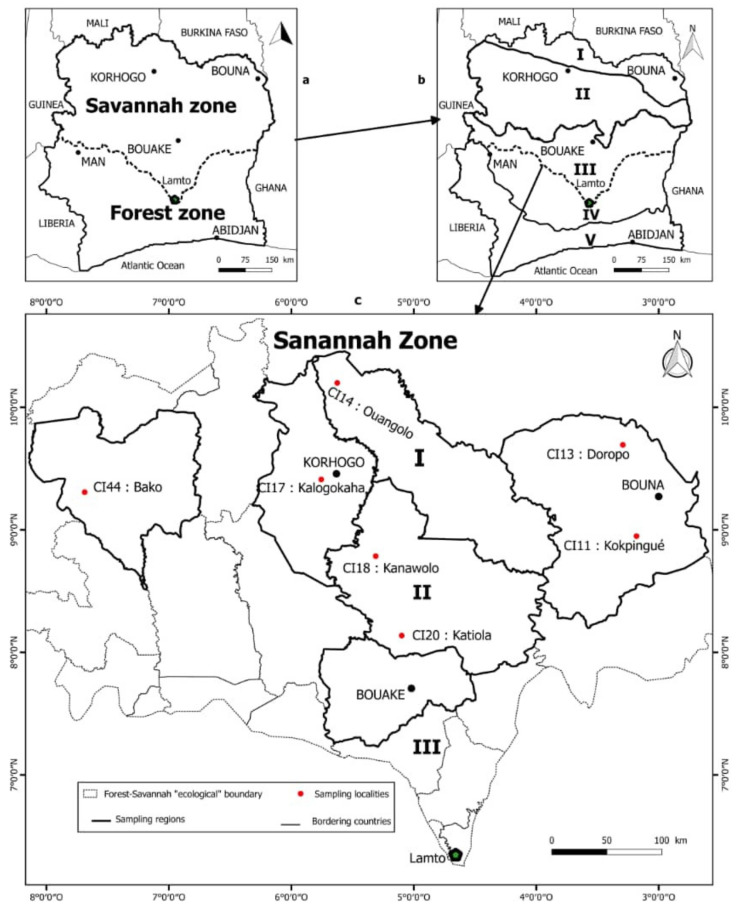
A map of Côte d’Ivoire with (**a**) the two large agro-ecological regions, i.e., the forest and savannah, both of which are divided (**b**) into five zones (I, II, III, IV and V) according to the vegetation types. (**c**) The different localities surveyed in the savannah zones in Northern Côte d’Ivoire are shown with red dots. Lamto is an ecological center for studying the tropical savannah in West Africa (http://lamto.free.fr/; accessed on 4 October 2020).

The study was carried out in the context of the African Soil Microbiome (AfSM) project, and the studied area was located in the savannah zones in Northern Côte d’Ivoire. Côte d’Ivoire is divided roughly into two large agro-ecological regions, of which the northern savannah region, where food crops, cotton and livestock predominate, and the fertile forest zone of the south, where most of the country’s cash crops are produced [50]. The boundary that marks the transition from forest to savannah is remarkably irregular (Figure 1a). It is characterized by the presence of an inverted triangular-like structure known as the « V-baoulé » (see [51,52]). Historically, the country was also divided into five zones according to the vegetation types, including (from the far north to the south) the Sudan savannah (I), the Sub-Sudan savannah (II), the Guinean savannah (III), the Semi-deciduous moist forest (IV) and the Evergreen moist forest (V) [46,53,54] (Figure 1b).

The study area covered the Sudanian savannah (I) and the Sub-Sudanian savannah (II) zones (Figure 1c). The annual rainfall is among the lowest in the country [53,55], ranging approximately from 1000 to 1750 mm per year [52,53,55]. The Sub-Sudanian savannah and the Sudanian savannah zones are also characterized by an average annual humidity of 60–70%, annual mean temperature of 24–27 °C and ferralitic and ferruginous soils [55,56]. The vegetation consists of grasslands, wooded grasslands and gallery forests [46,56]. Narrow gallery forests extend along watercourses and drainage basins, where very tall trees, such as *Ceiba pentandra*, *Sterculia tragacantha* and *Triplochiton scleroxylon,* dominate. The dominant tree species of the wooded grasslands are *Acacia albida*, *Khaya senegalensis*, *Parkia biglobosa,* and *Tamarindus indica*, and herbaceous plants include *Andropogon tectorum* and *Pennisetum purpureum*. Dominant trees in savannahs consist also of *Butyrospermum parkii, Daniellia oliveri* and *Lophira lanceolata*, as well as *Andropogon ivorensis, Loudetia simplex* and *Panicum phragmitoides* for herbaceous species [53,56]. As for the cultivated fields, they consist mainly of cashew trees and cereals (maize and rice). The sampled soils belong to the rhizosphere of all the vegetation types we described (Table 1).

### 2.2. Soil Sampling

Soil samples were collected in August-September 2017 from seven sites located in five administrative regions (Table 1) and alongside the national roads. Each sampled soil belongs to the rhizosphere of a natural herbaceous or wooded vegetation and/or a cultivated plant species (cashew, maize, rice etc.) (Table 1). The distance between sampling sites spanned 50–300 km. Each sampling site was represented by an area of approximately 100 m × 50 m with four independent sample locations (a virtual 1 m^2^ quadrat) at the corners of the oblong (Figure S1). At each of the four independent sample locations, four topsoil cores (2 cm in diameter and 5 cm in depth) (pseudo-replicate samples) were collected, pooled together, and homogenized into a composite sample of approximately 25 g (replicate sample). Four independent replicate samples (4 × 25 g) obtained from four sample locations at each sampling site were kept in a labelled sterile plastic bag and formed an independent soil sample. This process was repeated for all seven sampling sites. In total, seven independent soil samples were obtained. Each soil sample taken from the two savannah zones from northern Côte d’Ivoire (CI) is referred to by the soil numbers 11, 13, 14, 17, 18, 20 or 44 (Table 1). After sampling, the soil samples were transported to the laboratory, where they were stored at 4°C prior to a shipment to South Africa for further analysis. 

### 2.3. Analysis of Soil Physicochemical Properties

The analysis of soil physico-chemical characteristics was carried out by Bemlab (Strand, Cape Province, South Africa) using standard methods. Briefly, prior to analyses, the samples were air-dried at room temperature for four days, separated from roots and debris, and passed through a 2 mm sieve. The sieved replicate samples of each sampling site were subsequently pooled together to obtain a composite soil sample. Physical characteristics (fractions of clay, sand and silt) were analyzed using the Bouyoucos sedimentation method (hydrometer method) [57]. The classification of soils according to texture was based on the standard USDA particle-size classification using the Soil Texture online Calculator (https://www.nrcs.usda.gov/wps/portal/nrcs/detail/soils/survey/?cid=nrcs142p2_054167; accessed on 8 April 2021). The pH (aqueous) was measured as described [58], while the oxidizable carbon content was determined using the dichromate oxidation method (the Walkley–Black method) [59]. Soil chemical parameters (exchangeable and soluble Na, K, Ca, Mg, Al, Fe, Mn and P) were analyzed using the Mehlich No. 3 soil test extractant with Inductively Coupled Atomic Emission Spectrometry (ICP-AES) procedures [60].

### 2.4. DNA Extraction, PCR, MiSeq Sequencing, and Sequence Data Analysis

Genomic DNA (gDNA) extraction, amplification and high-throughput amplicon sequencing were carried out as in Nkuekam et al. [61], with few modifications. DNA extraction was conducted at the Centre for Microbial Ecology and Genomics (University of Pretoria, South Africa). Briefly, soil samples were first ground with Powerlyser (Mo Bio Laboratories Inc.), and the genomic DNA was extracted from 0.25 g of soil using the PowerSoil DNA isolation kit (Mo Bio Laboratories Inc., Carlsbad, CA, USA). The success of the extraction was verified by 1% agarose gel electrophoresis visualizing under UV light. DNA amplification was conducted at the MRDNA sequencing facility (www.mrdnalab.com, accessed on 25 June 2021, Shallowater, TX, USA) in a 30-cycle PCR using the HotStarTaq Plus Master Mix Kit (Qiagen, Germantown, MD, USA) [61]. The V4–V5 variable region of the 16S rRNA gene was amplified and sequenced using the alternative forward primer 515F-Y (5′-GTGYCAGCMGCCGCGGTAA-3′; [62]) and the universal reverse primer 909–928 (5′-CCCCGYCAATTCMTTTRAGT-3′; [63]), with 12 nucleotides unique barcode at 5-end of 515F-Y for each soil sample. High-throughput amplicon sequencing was performed using an Illumina MiSeq platform at the MRDNA sequencing facility.

For the processing of the sequencing data, raw sequences were first checked for reads quality using FastQC (https://www.bioinformatics.babraham.ac.uk/projects/fastqc/; accessed on 1 May 2021). Reads were then sorted based on unique soil sample tags using Sabre 1.0 program (https://github.com/najoshi/sabre; accessed on 1 May 2021) with default parameters and trimmed for primer and barcode removal using cutadapt 2.10 [64]. The trimmed sequences were subsequently denoised using the DADA2 algorithm [65] that resolves Illumina-sequenced amplicon errors to generate amplicon sequence variants (ASVs). ASVs were classified using the RDP classifier [66] with default parameters. The assignment of ASVs to rhizobia taxa was processed through a search for similar sequences conducted with BLAST v. 2.9.0+ [67] against the SILVA 138.1 database [68].

The taxonomic affiliations obtained with SILVA was manually validated at the genus level using several approaches, including (i) BlastN with NCBI/GenBank online standard databases (nucleotide collection (nr/nt) and Whole-genome shotgun contigs (wgs)) to select closely related reference sequences, (ii) phylogenetic reconstructions and (iii) similarity level calculations between the ASVs and selected rhizobial reference sequences. Briefly, V4-V5 edited sequences were aligned with MUSCLE as implemented in MEGA software v. 7 and phylogenies were inferred subsequently with evolutionary trees reconstructed using the maximum likelihood (ML) and neighbor-joining (NJ) methods [69,70]. Best-fit nucleotide substitution models were selected according to the Bayesian information criterion (BIC) [71] and the uncorrected genetic distances calculated as in Rashid et al. [72]. Phylogenetic analyses also included 16S rRNA gene sequences derived from archived genome data of the type species of all the 18 alphaproteobacterial and betaproteobacterial genera harboring rhizobia isolates [15], except for *Paraburkholderia. P. graminis* PHS1 16S rRNA gene data was used in the analysis as a surrogate of *P. graminis* type strain C4D1M, for which no full rRNA gene sequence was accessible at the time of writing (June 2021). Details of the type species of all the 18 bacterial genera harboring rhizobia are listed in Table 2.

### 2.5. Statistical and Diversity Index Analyses

The statistical and diversity index analyses were performed using R v. 4.0.3 [95], including the R packages vegan [96], phyloseq [97] and ggplot2 [98]. The rarefaction curves were computed using the vegan function rarefy, which is based on Hurlbert’s formula [99] to evaluate the sequencing efforts provided. As a normalization step to reduce bias associated with different sequencing depths, all samples were subsampled down to the size of the smallest sample. Each sample was rarefied to 1384 reads. Indices of richness (Chao1) and alpha diversity (Shannon, Simpson and Fisher) were calculated by savannah zone, and a non-parametric Wilcoxon test was used to compare the mean values at the significance level of 5%. The degree of community differentiation (beta-diversity) was evaluated to calculate Jaccard’s similarity coefficient and the Bray–Curtis index of (dis)similarity for each ASV. The relationship between the ASVs of rhizobia and the environmental parameters that characterize the soils of the savannah zones of Northern Côte d’Ivoire was assessed by canonical correspondence analysis (CCA) using ten physico-chemical parameters. Prior to drawing the relative abundance of rhizobia taxon per soil sample, the sample counts of ASVs were used to calculate relative abundance by computing the ratio of the count of each sample by the sum of the counts of all samples. The obtained relative abundance of counts was used to draw the bar plot of relative abundance of ASVs by genera and family between samples. The heatmap was created using the ecologically organized plot_heatmap function of the phyloseq package, which is a variant of the heatmap provided by the NeatMap package [100]. To draw the heatmap, we used the NMDS ordination method and the Bray–Curtis distance. The 16S rRNA gene sequences used in this study are available in the NCBI SRA database under accession number SRR13623326 (CI11), SRR13623324 (CI13), SRR13623323 (CI14), SRR13623320 (CI17), SRR13623319 (CI18), SRR13623317 (CI20) and SRR13623335 (CI44).

### 2.6. In Silico Evaluation of the 16S rRNA Gene V-Regions Discriminatory Power for Rhizobia

The aim of this analysis was to compare in silico the discriminatory power at the genus level of nine commonly used 16S rRNA gene V-regions for rhizobia. Prior to this analysis, we estimated the current number of species of rhizobia. We counted the number of species of rhizobia validly published within the 18 alphaproteobacterial and betaproteobacterial genera harboring rhizobial species and provided on the List of Prokaryotic names with Standing in Nomenclature (LPSN), also known as bacterio.net [101]. We also documented the prevalence of species with nodulation (Nod) and/or N_2_-fixation (Fix) capacities using the original publications describing novel taxa of rhizobia and accessible on the LPSN website, as well as the more recent publications that reviewed the symbiotic features of rhizobial taxa [102,103,104]. All the data are reported in Table 2. As for the evaluation of the discriminatory power of V-regions, nine V-regions (V1 to V9) spanning the entire 16S rRNA gene and commonly targeted in microbial metagenomic analyses were selected [63,105,106,107] (Table 3). The corresponding universal primers targeting the selected V-regions and their relevant characteristics are reported in Table 3. The V-regions were compared to the full-length size of the 16S rRNA gene sequences with a method used in VanInsberghe et al. [108]. Briefly, the full-length 16S rRNA gene sequences were aligned for all the 18 genera with MAFFT version 7 using the Q-INS-I method [109] and followed by a maximum likelihood phylogenetic tree reconstruction as well as by a pairwise similarity distances calculation as in Rashid et al. [72]. Similarity values were used to identify the uniquely distinguishable taxa at 97%, 99% or 100% cut-offs. Subsequently, the full-length 16S rRNA gene sequence alignment was edited to the total number of positions that corresponds to those of each V-region, in addition to that of the V1-V9 region, which is the near-full-length size of the 16S rRNA gene (Table 3). The total number of positions in each edited dataset was used to calculate the similarity values that served to identify the uniquely distinguishable taxa for the given V-region.

## 3. Results

We carried out a high-throughput amplicon sequencing (HTAS) analysis of the 16S rRNA gene V4-V5 region to assess the taxonomy, diversity and distribution of rhizobial taxa in seven soils in the Sudan savannah (I) and the Sub-Sudan savannah (II) zones in Northern Côte d’Ivoire (Figure 1). These two zones have been largely neglected in terms of fundamental research in microbial ecology, and this study provides their first comprehensive rhizobial microbiome analysis. The sampled soils from the two zones were analyzed for their physico-chemical properties prior to the HTAS analysis. 

### 3.1. Physico-Chemical Properties of Soil Samples 

The soils’ physico-chemical data are reported in Supplementary Table S1. The seven studied localities have similar soil textures characterized by a high proportion of sand (>70%) but can be divided into two subgroups: soils CI13, CI14, CI17, CI18 and CI20 were sandy loams, while CI11 and CI44 were loamy sand. The pH of the seven soils ranged from 5 to 7, being consistent with the soil pH range expected in tropical humid regions (https://www.qld.gov.au/environment/land/management/soil/soil-properties/ph-levels; accessed on 8 April 2021). Soils CI11 and CI13, both of which were from the locality of Bouna in the north-east (Figure 1), were neutral (pH 6.6), while CI14, CI17, CI18, CI20 and CI44 soils were acidic (pH < 6.5). CI14 (pH = 6.4) was the least acidic soil (nearly neutral). The distribution of the soil samples according to the chemical properties was more heterogeneous. CI11 was among the soil samples having the highest values of calcium (Ca^2+^), magnesium (Mg^2+^), potassium (K^+^) and sodium (Na^+^), while CI44 and CI-17 had the lowest values for the same mineral elements.

### 3.2. Sequence Data and Taxonomic Affiliation

**Figure 2:**
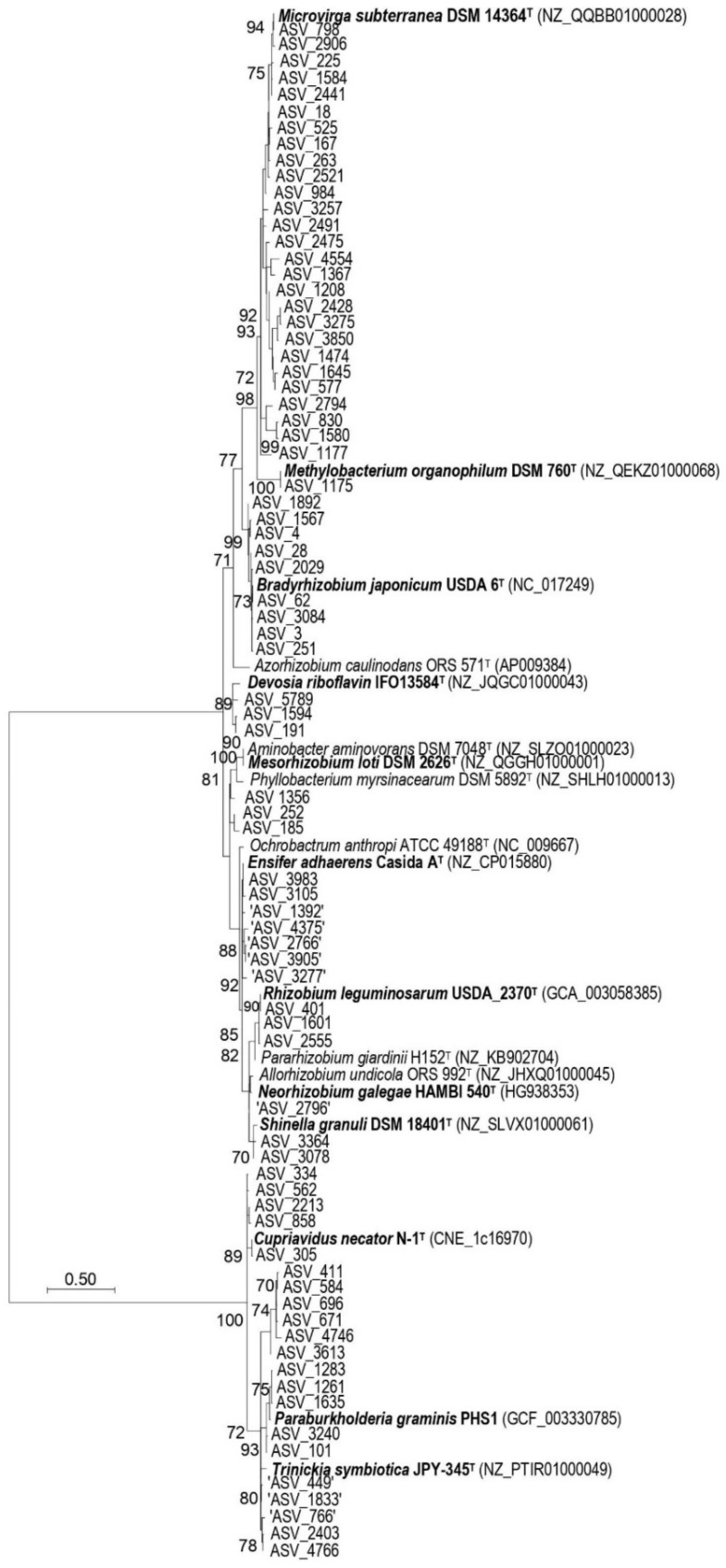
An unrooted phylogenetic tree of ASVs of rhizobia detected in savannah soils of Northern Côte d’Ivoire using the 16S rDNA V4-V5 variable region with all the type species of 18 *Alpha*- and *Beta*-*proteobacteria* genera harboring described rhizobia species. Evolutionary relationships were inferred using the Maximum Likelihood method based on the Tamura 3-parameter using a discrete Gamma distribution with invariant sites (T92+G+I). Bootstrap values ≥ 70% based on 1000 replicates are indicated, and the scale bar represents the number of substitutions per site. Type species of the 18 genera are displayed with strain ID followed by the GenBank 16S rRNA gene accession number. DNA sequences for ASVs used in this tree are provided in Table S3, and they are taken from the complete sequencing data archived in the NCBI SRA database. All ASVs that could not be accurately identified in the tree are enclosed in quotation marks.

The amplification of the total DNA extracted from the seven soil samples using V4-V5 primers yielded ca. 400–500 nucleotide length products, as expected for bacteria (Table 3). The rarefaction curves reach the asymptote with less than 1000 sequences, suggesting that the sequencing effort of each amplicon was sufficient (Figure S2). From a total of 900,760 sequences obtained through Illumina’s high throughput sequencing platform, a total of 786,283 sequences were considered for the clustering after sequence trimming. When clustered and quality-controlled, the 786,283 sequences yielded 5997 ASVs in total, of which 80 (less than 2%) matched to rhizobia in the SILVA database. This assignment of the ASVs to rhizobia taxa was further refined using a multi-step approach that includes phylogenetic analyses of the ASVs (Figure 2; Figure S3) as well as a genetic distance comparison (Table S2) and online blastN analyses. Phylogenetic assignments of the ASVs performed with a subset of 86 closely related sequences (99 to 100% similar) and/or 18 type species of Proteobacteria harboring rhizobia species validly published to date yielded similar taxonomic affiliations (see Figure 2 and Figure S3, respectively). Together, these different analyses improved the taxonomic identification, with 77 ASVs (equivalent to 15,886 sequences) being confirmed as rhizobia (Figure 2; Table S3; Table S4). The 77 ASVs belonged to 12 genera of rhizobia (*Bradyrhizobium*, *Cupriavidus*, *Devosia*, *Ensifer*, *Mesorhizobium*, *Methylobacterium*, *Microvirga*, *Neorhizobium*, *Paraburkholderia*, *Rhizobium*, *Shinella* and *Trinickia*) (Figure 2) and were present in six families (*Burkholderiaceae*, *Devosiaceae*, *Methylobacteriaceae*, *Nitrobacteraceae*, *Phyllobacteriaceae* and *Rhizobiaceae*) of the classes *Alphaproteobacteria* (09 genera) and *Betaproteobacteria* (03 genera) (Table S3). In silico taxonomic assignments of the 77 ASVs revealed that many families in the class *Alphaproteobacteria* such as *Bradyrhizobiaceae* (renamed *Nitrobacteraceae*), *Methylobacteriaceae* or *Phyllobacteriaceae* are not accurately assigned in SILVA 138 database, as reported elsewhere [114]. Indeed, these three families were misidentified, including *Xanthobacteraceae, Beijerinckiaceae* and *Rhizobiaceae*, respectively (Table S2). These weaknesses were compensated using the validly published names reported on the LPSN website [101].

Of the 12 genera detected, *Microvirga* (24 ASVs), *Paraburkholderia* (11 ASVs) and *Bradyrhizobium* (9 ASVs) are the most dominant, following the criteria of the number of ASVs detected per genus (Table S5). These three genera represented more than 57% of the total rhizobial ASVs (Table S5). Of the 18 *Alpha*- and *Beta-proteobacterial* genera harboring described rhizobial species, those not detected in this analysis included *Allorhizobium*, *Aminobacter*, *Azorhizobium*, *Ochrobactrum*, *Pararhizobium* and *Phyllobacterium*. Interestingly, these six genera have a low relative number of validly published species (only 11%) (Table 2).

### 3.3. Relative Abundance of Rhizobia Taxa per Soil Sample

**Figure 3:**
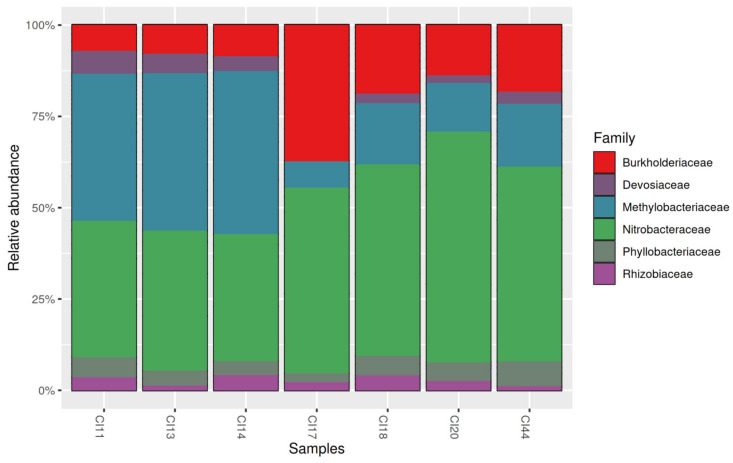
The relative abundance of the six families of rhizobia in northern Côte d’Ivoire savannah soils. CI11: soil from the locality of Kokpingué; CI13: Doropo; CI14: Ouangolo; CI17: Kalogokaha; CI18: Kanawolo; CI20: Katiola; CI44: Bako.

Relative abundance was expressed as a percentage with respect to the total number of sequences in each soil sample. The analysis of relative abundance showed that *Nitrobacteraceae* (formerly *Bradyrhizobiaceae*) was by far the most abundant taxon (Figure 3).

**Figure 4:**
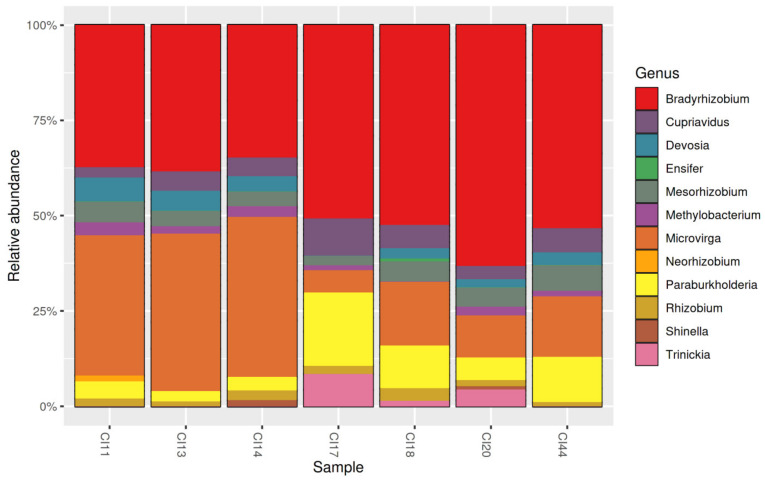
The relative abundance of the 12 genera of rhizobia detected from the savannah soils in Northern Côte d’Ivoire. CI11: soil from the locality of Kokpingué; CI13: Doropo; CI14: Ouangolo; CI17: Kalogokaha; CI18: Kanawolo; CI20: Ka-tiola; CI44: Bako.

At the genus level, *Bradyrhizobium* (*Nitrobacteraceae*), *Microvirga* (*Methylobacteriaceae)* and *Paraburkholderia* (*Burkholderiaceae*) were the most abundant taxa (Figure 4), where the cumulative relative abundance of these three genera across all soil samples represented ca. 80% of all sequences, as follow: *Bradyrhizobium* (49.1%), *Microvirga* (21.4%) and *Parabukholderia* (9.0%) (Table S5). The two least prevalent genera were *Neorhizobium* (0.19%) and *Shinella* (0.31%), being detected in only one and two soils, respectively (Figure 4; Table S5).

**Figure 5:**
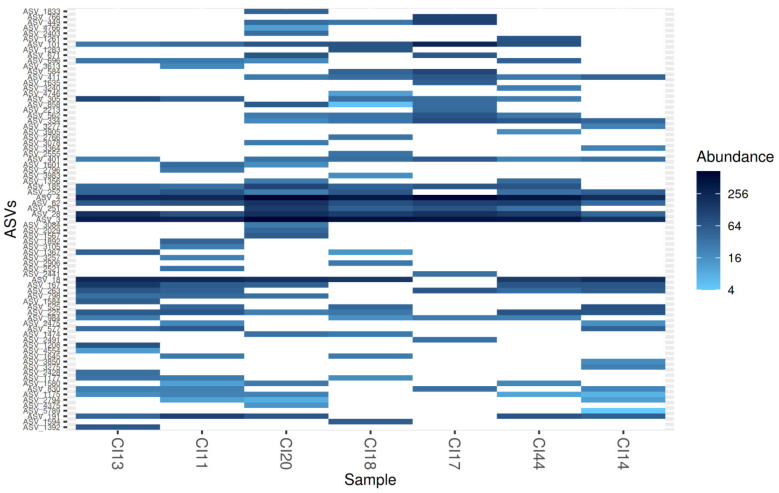
A heat map illustrating the relative abundance and the ubiquity of each of the 77 ASVs detected from the savannah soils in Northern Côte d’Ivoire.

Of the 77 ASVs, two were highly abundant (>10%); namely ASV_3 (17. 8%) and ASV_4 (17. 7%) (Table S3). ASV_3, ASV_4, ASV_28 and ASV_62, all of which belonged to the genus *Bradyrhizobium* genus, were prevalent in all soil samples (Figure 5). Six of the 12 rhizobial genera detected in this study were ubiquitous in the savannah soils of Northern Côte d’Ivoire. They included *Bradyrhizobium*, *Cupriavidus*, *Mesorhizobium*, *Microvirga*, *Paraburkholderia* and *Rhizobium* (Figure 4; Table S5).

### 3.4. Richness and Diversity Indices

The seven soils had a comparable number of ASVs (ASVs richness) which ranged from 24 (soil # CI11 and # CI17) to 35 (# CI20) (Table S5). The indices of richness (Chao1) and alpha diversity (Shannon, Simpson and Fisher) analyzed per savannah biome are similar among the Sudanian savannah and the Sub-Sudanian savannah (Table S6). The community diversity indices showed that the sites CI18 and CI44 shared the lowest value of Bray-Curtis dissimilarity (calculated value of 0.29), meaning that these two sites shared the highest number of ASVs together when the composition of all the seven sites was compared. In contrast, CI14 and CI17 had the lowest number of shared ASVs (Table 4).

The Jaccard distance from the community diversity analysis revealed that the sites CI11 and CI17 were the most dissimilar (Jaccard distance of 0.82) while CI44 and CI20 were the least dissimilar among all the seven sites (Jaccard distance of 0.52) (Table 5).

**Figure 6:**
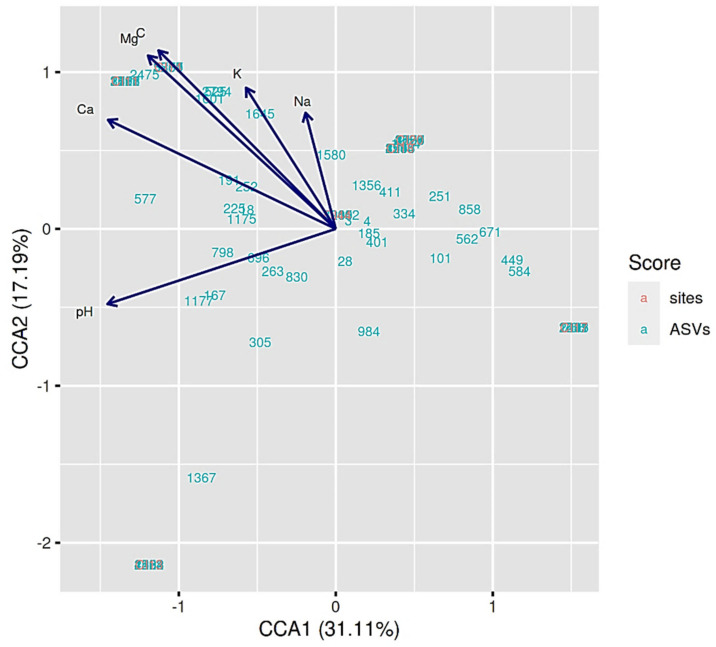
Canonical correspondence analysis (CCA) showing the relationship between the 77 ASVs of rhizobia (represented by their number) and the physico-chemical parameters of soils from Northern Côte d’Ivoire. The arrows represent soil properties.

The canonical correspondence analysis showed that the pH, C, Ca^2+^, K^+^, Mg^2+^ and Na^+^ were the soil properties that most strongly influenced the distribution of rhizobial taxa from the savannah soils in Northern Côte d’Ivoire **(**Figure 6).

### 3.5. In Silico Evaluation of 16S rDNA V-Regions Discriminatory Power for Rhizobia

**Figure 7:**
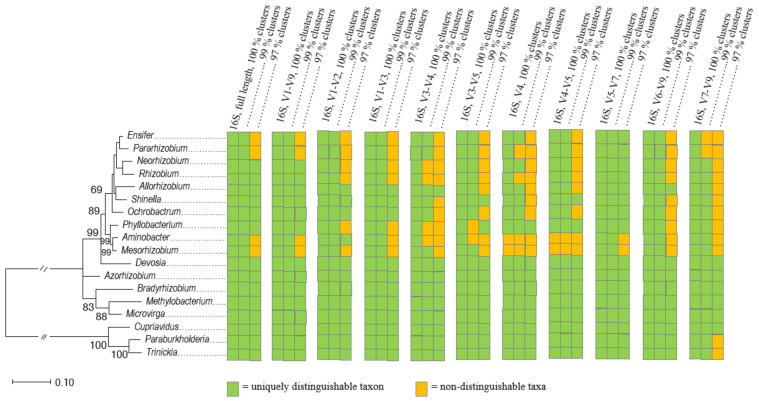
An unrooted phylogenetic tree based on the full-size sequence of the 16S rDNA (1564 positions), using the Maximum Likelihood method and Tamura-Nei model using a discrete Gamma distribution with invariant sites (TN93+G+I). Bootstrap values ≥ 70% based on 1000 replicates are indicated, and the scale bar represents the number of substitutions per site. A green shaded box indicates that a taxon can be uniquely distinguished with the given V-region and gene length and clustering method, while an orange box indicates that a taxon is merged with at least one other taxon in at least one gene cluster.

In an attempt to assign the 77 rhizobial ASVs detected from the savannah soils in Northern Côte d’Ivoire to the 18 genera of rhizobia validly published to date, we found that some genera type species, including *Aminobacter aminovorans* DSM 7048^T^ and *Mesorhizobium loti* DSM 2626^T^, had identical 16S rRNA gene V4-V5 region (Figure 2). Thus, we struggled to cluster at the genus level all the ASVs that belong to the *Aminobacter - Mesorhizobium* clade, including ASV_185, ASV_252 and ASV_1356. These weaknesses were compensated using several approaches, including BlastN with NCBI/GenBanK online nr/nt and wgs databases. However, the taxonomic affiliation of nine ASVs that belonged to the clades of *Allorhizobium*-*Neorhizobium*-*Pararhizobium*-*Rhizobium* and *Burkholderia*-*Caballeronia*-*Parabukholderia* was partially solved (Figure 2, Table S2). In silico comparisons of different 16S rRNA gene variable regions to assess their effectiveness for differentiating rhizobia confirmed that the V4-V5 region has an insufficient resolution for separation of all genera of rhizobia at the genus level. As expected [115], the V4 region alone did also not perform well, regardless of the threshold used for the delineation. Both V4 and V4-V5 primer pairs were the only sets of primers that could not discriminate all 18 genera at the ASV level (one nucleotide polymorphism level). In contrast, the analysis showed that the V5-V7 region was the best target for genus discrimination (Figure 7): the V5–V7 region discriminated all the 18 genera of rhizobia at 100% and 99% thresholds, and 16 genera at the 97% threshold (Figure 7). It is noted that the two genera which were not discriminated by the V5-V7 region at the 97% threshold were *Aminobacter* and *Mesorhizobium*, suggesting that a threshold higher than the classical 97% should be used when targeting these two genera in HTAS analyses.

## 4. Discussion

A few studies reporting a characterization of soil rhizobial communities using the HTAS of 16S rDNA variable regions were carried out in temperate arable soils in East Europe [45] and coniferous forest soils in North America [41]. Up to now, little is known of the soil microbiome of semiarid areas commonly known as savannahs [116,117], although there are considered important biodiversity hotspots, including for microorganisms [118,119]. Two examples of savannah biomes have been neglected in terms of research in microbial ecology for decades: the Brazilian Cerrado savannah and the African savannah [116,120,121]. The microbiome of the Cerrado savannah is relatively more explored, including for Archaea [122], Bacteria [118,123], Fungi [124] and Protists [125], unlike that of the African savannah, which has not been studied in a systematic manner [35,48,126]. The current study is among the pioneer studies on African savannahs microbiome [35,127], and it provides new insights into the presence and distribution of taxa of rhizobia across the Sudanian and the Sub-Sudanian savannah zones. It revealed that the rhizobial diversity in the savannah zones in Northern Côte d’Ivoire is considerable in terms of richness and relative abundance of genera and families detected. These findings are similar to those observed in the Brazilian Cerrado savannah [118,123] and the African Miombo Woodlands in Mozambique [127], where rhizobacteria, including rhizobia, were found genetically diversified and abundant [118,127]. However, these results contrast with a similar study carried out in the Mopane woodlands, another important savannah ecosystem in southern Africa [35].

Of the 18 *Alpha*- and *Beta-proteobacterial* genera harboring the described rhizobial species, only *Allorhizobium*, *Aminobacter*, *Azorhizobium*, *Ochrobactrum*, *Pararhizobium* and *Phyllobacterium* were not detected in soils from the savannah zones in Northern Côte d’Ivoire. As these six genera have also a low relative number of validly published species to date, all these data suggested that they are probably less abundant and diversified in soils and/or are associated with a limited set of legumes species. From all the 12 genera detected, *Bradyrhizobium* was found more abundant and ubiquitous, together with *Microvirga* and *Paraburkholderia*. Contrasting findings have been reported on the prevalence, genetic diversity and the ubiquity of these three genera of rhizobia. An HTAS study of the potential nitrogen-fixing bacteria in Polish soils detected *Devosia*, *Mesorhizobium*, *Methylobacterium, Microvirga*, *Phyllobacterium*, and *Rhizobium* (alpha-rhizobia), as well as *Burkholderia sensu lato (s.l.)* and *Cupriavidus* (beta-rhizobia), but noted the absence of *Bradyrhizobium* [45]. In contrast to Wolińska et al. [45], a recent atlas established for dominant soil bacteria classified *Bradyrhizobium* and *Devosia* among the most abundant and ubiquitous bacteria worldwide, with an apparent paucity of *Burkholderia s.l* in soils [6]. Nevertheless, a survey of the top 20 most abundant genera found in soil samples revealed that *Bradyrhizobium* and *Burkholderia s.l.* are, respectively, the first and the second most prevalent genera of soil bacteria [128]. Although all these data indicated that *Bradyrhizobium* and/or *Burkholderia s.l.* and/or *Microvirga* were frequently detected among the most dominant bacteria genera in soil samples, to our knowledge, the current study is the first showing that the *Bradyrhizobium* genus dominates in tropical savannah soils, together with *Microvirga* and *Paraburkholderia*. The predominance and the ubiquity of rhizobia genera, including *Bradyrhizobium* and *Burkholderia s.l*., is thought to be due to their genetic diversity, and their catabolic versatility that enables them to degrade recalcitrant compounds and survive in oligotrophic environments [41,129,130]. Since Moulin et al. [131] described two *Burkholderia* nodule-forming strains isolated in French Guiana and in South Africa, beta-rhizobia have been routinely identified from soils, mainly in South Africa, South America and southeast Asia [40]. Some of these studies even reported the dominance of *Paraburkholderia* when compared to cosmopolitan *Bradyrhizobium* in several soils, depending on the biome (e.g., the Cerrado, Caatinga and Forest Atlantic biomes in Brazil; the Fynbos biome in South Africa), the legumes species (*Mimosa* spp.; *Lebeckia* spp.) and the soil types [39,40,104,132]. Several studies have demonstrated that the beta-rhizobia are well adapted to poor and acidic soils [37,133,134]. Our study suggests that *Paraburkholderia* and *Trinickia* are more abundant in the mildly acidic soils (pH 5.7 < pH< 6.0), all of which harbored anthropogenic activities (fields of cashew and cereals etc.). Despite this observation in the cultivated soils, the impact of the savannah types on the dynamics of rhizobia diversity and abundance was not established in this study.

Although the variable regions of the 16S rRNA gene (e.g., individual V-regions, adjacent V-regions, pairs of non-contiguous V-regions) are well known [106,135,136], the selection of the most efficient variable region (s) for microbiome analysis is still debated [63,106,107,110,137,138,139]. Many studies indicated that the efficiency of the variable regions for HTAS analysis depends on multiple parameters, including the microorganisms of interest and the extent to which their 16S rRNA genes have evolved [105,140,141]. For rhizobia, our study suggested that the V5-V7 region could be suitable for differentiating strains at the genus level, possibly replacing the use of the V4-V5 region. In a previous study, Eardly et al. [142] identified the V7 region alone as highly polymorphic in the Rhizobiales. Taken together, we suggest that the V5-V7 region contains sufficiently polymorphic DNA sequences to resolve the genetic complexity of the full 16S rRNA gene in rhizobia.

Many studies had reported the use of single-copy housekeeping genes in microbiome analyses to improve resolution at species and subspecies levels [143,144,145,146,147]. A multigenic approach that includes at least one housekeeping gene (e.g., *rpoB*) and one variable region of the 16S rRNA gene [148] is also considered a promising methodology. Taking into account these recommendations, we further propose the use of the V5-V7 region to analyze the rhizobial microbiome in combination with one of the four housekeeping genes (*atpD*-*gyrB*-*recA*-*rpoB*) that have been used for resolving ambiguous cases of identification among *Rhizobium* strains [149].

## Figures and Tables

**Table 1:** The geographic positions and main characteristics of the sampled soils.

Soil ID	GPS Location	Location (Province/City/Region)	Environment
CI11	N08°58′52.1”, W003°10′50.1”	Kokpingué/Bouna/Bounkani	Natural wooded grassland soils (*Acacia* spp.)
CI13	N09°41′49.9”, W003°17′43.9”	Doropo/Bouna/Bounkani	Natural wooded grassland soils (*Parkia biglobosa*)
CI14	N10°04′54.6”, W005°24′41.0”	Ouangolo/Ouangolo/Tchologo	Rice field, Native Forest & natural grassland soils
CI17	N09°13′11.1”, W005°35′22.0”	Kalogokaha/Korhogo/Poro	Cashew field soils
CI18	N08°52′12.5”, W005°22′08.0”	Kanawolo/Niakara/Hambol	Cashew field and natural grassland soils (*Loudetia* spp.)
CI20	N08°05′38.2”, W005°05′01.3”	Katiola/Katiola/Hambol	Maize & cassava fields and natural grassland soils
CI44	N09°03′41.5”, W007°35′20.8”	Bako/Odienné/Denguélé	Cashew field and woodeed savanna soils

**Table 2:** A list of the 18 alpha-and beta-proteobacterial genera harboring rhizobia, their type species used in this study and their corresponding relevant characteristics.

No.	Genus	Number of Species ^3^	Number of Nod+/ Fix+ species ^4^	Genus Type Species	Genome Accession	16S rRNA Gene Full Size (bp) ^6^	Symbiotic Capacity of the Genus Type Species
1	*Allorhizobium*	8	1	*Allorhizobium undicola* ORS 992^T^	NZ_JHXQ01000045	1482	Nod+/Fix+ [73]
2	*Aminobacter*	7	1	*Aminobacter aminovorans* DSM 7048^T^	NZ_SLZO01000023	1484	unknown [74]
3	*Azorhizobium*	3	2	*Azorhizobium caulinodans* ORS 571^T^	AP009384	1482	Nod+/Fix+ [75]
4	*Bradyrhizobium*	57	55	*Bradyrhizobium japonicum* USDA 6^T^	NC_017249	1488	Nod+/Fix+ [23]
**5**	***Cupriavidus ^1^***	**18**	**2**	***Cupriavidus necator* N-1^T^**	**CNE_1c16970**	**1531**	Nod−^7^ [76]
6	*Devosia*	26	1	*Devosia riboflavina* IFO13584^T^	NZ_JQGC01000043	1481	*nod*/*fix* genes were not detected [77]
7	*Ensifer*	20	16	*Ensifer adhaerens* Casida A^T^	NZ_CP015880	1484	Nod−, *nod* genes were not detected [78]
8	*Mesorhizobium*	56	45	*Mesorhizobium loti* DSM 2626^T^	NZ_QGGH01000001	1484	Nod+/Fix+ [79,80]
9	*Methylobacterium*	45	1	*Methylobacterium organophilum* DSM 760^T^	NZ_QEKZ01000068	1482	unknown [81]
10	*Microvirga*	17	5	*Microvirga subterranea* DSM 14364^T^	NZ_QQBB01000028	1486	unknown [82]
11	*Neorhizobium*	4	4	*Neorhizobium galegae* HAMBI 540^T^	HG938353	1480	Nod+/Fix+ [83,84]
12	*Ochrobactrum ^2^*	17	2	*Ochrobactrum anthropi* ATCC 49188^T^	NC_009667	1482	non-symbiotic bacterium [85,86]
**13**	***Paraburkholderia ^1^***	**73**	**16**	***Paraburkholderia graminis* PHS1 ^5^**	**GCF_003330785**	**1532**	unknown [87,88]
14	*Pararhizobium*	6	2	*Pararhizobium giardinii* H152^T^	NZ_KB902704	1484	Nod+/Fix+ [89,90]
15	*Phyllobacterium*	12	3	*Phyllobacterium myrsinacearum* DSM 5892^T^	NZ_SHLH01000013	1484	Nod− [91]
16	*Rhizobium*	91	48	*Rhizobium leguminosarum* USDA 2370^T^	GCA_003058385	1480	Nod+/Fix+ [27]
17	*Shinella*	8	1	*Shinella granuli* DSM 18401^T^	NZ_SLVX01000061	1484	unknown [92]
**18**	***Trinickia ^1^***	**7**	**1**	***Trinickia symbiotica* JPY-345^T^**	**NZ_PTIR01000049**	**1530**	Nod+/Fix+ [76]

^1^ In bold: belong to the class of *Betaproteobacteria* (these three genera have the largest 16S rRNA gene size among the 18 genera of rhizobia). ^2^ *Brucella anthropi* (Holmes et al., 1988) is now proposed as comb. nov. [basonym: *Ochrobactrum anthropi* Holmes et al. 1988] [20,85,93]. ^3^ Number of species with a validly published and correct name according to the List of Prokaryotic names with Standing in Nomenclature (LPSN) database, accessed on 9 October 2020. ^4^ Species nodulation (Nod) and N_2_-fixation (Fix) capacities according to publications accessed directly via the LPSN website and additional references (See Material and Methods, Section 2.6): ca. 43% of species nodulated. ^5^ *Paraburkholderia graminis* PHS1 was used as a surrogate of *P. graminis* C4D1M^T^, for which no full 16S rRNA gene sequence was accessible at the time of writing (June 2021). ^6^ The 16S rRNA gene size was determined using the annotation of *Escherichia coli* K-12′s 16S rDNA (genome accession number U00096). ^7^ The type strain does not have a symbiotic capacity, but many strains belonging to the same species were reported as Nod+/Fix+ [76,94].

**Table 3:** Nine commonly used 16S rDNA primers targeting the variable regions and the V1-V9 region and corresponding relevant characteristics used in this study.

16S rDNA V-region ^1^	Forward Name ^2^	Forward Sequence (5′ to 3′)	Reverse Name	Reverse Sequence (5′ to 3′)	Size (bp) Variation Among Rhizobia ^3^	Size variation OnceEdited (Primers Deleted)	Total Positions in the Final Dataset Aligned ^4^
V1-V9	27F	AGAGTTTGATCMTGGCTCAG	1492Rmod	TACGGYTACCTTGTTAYGACTT	1445–1497	1403–1455	1486
V1-V2	27F	AGAGTTTGATCMTGGCTCAG	337R	CYIACTGCTGCCTCCCGTAG	320–350	280–310	325
V1-V3	27F	AGAGTTTGATCMTGGCTCAG	534R	ATTACCGCGGCTGCTGG	468–523	431–486	501
V3-V4	341F	CCTACGGGNGGCWGCAG	805R	GACTACHVGGGTATCTAATCC	440–465	402–427	427
V3-V5	341F	CCTACGGGNGGCWGCAG	926Rb	CCGTCAATTYMTTTRAGT	560–585	525–550	550
V4	515F	GTGCCAGCMGCCGCGGTAA	806R	GGACTACHVGGGTWTCTAAT	292	253	253
V4-V5	515F–Y	GTGYCAGCMGCCGCGGTAA	909–928R	CCCCGYCAATTCMTTTRAGT	413	374	374
V5-V7	799F	AACMGGATTAGATACCCKG	1193R	ACGTCATCCCCACCTTCC	409–417	372–380	385
V6-V9	928F	TAAAACTYAAAKGAATTGACGGGG	1492Rmod	TACGGYTACCTTGTTAYGACTT	605–612	560-567	576
V7-V9	1100F	YAACGAGCGCAACCC	1492Rmod	TACGGYTACCTTGTTAYGACTT	408–415	371-378	380

^1^ For general information about the selected set of primers of each V-region, refer to references [63,105,106,110]. ^2^ Numbering based on the *Escherichia coli* 16S rRNA gene system of nomenclature [63,106]. ^3^ Determined in silico in this study using all 18 alphaproteobacterial and betaproteobacterial genera harboring rhizobial species. The 3′- and 5′- end conical structure of *E. coli* 16S rRNA gene described elsewhere [111,112], together with the annotation and numbering system of *Escherichia coli* K-12 (genome accession number U00096) [113], were used to delineate the full-length size of the 16S rRNA gene sequence. ^4^ Obtained with the 16S rRNA gene sequences (without primers sequences) of all the 18 genera of rhizobia aligned using Muscle as implemented in MEGA7.

**Table 4:** The measure of the beta-diversity as indicated by the Bray–Curtis distance.

Bray–Curtis Distance	CI11	CI13	CI14	CI17	CI18	CI20
CI13	0.30	-	-	-	-	-
CI14	0.33	0.40	-	-	-	-
CI17	0.65	0.58	0.67	-	-	-
CI18	0.46	0.46	0.51	0.41	-	-
CI20	0.52	0.49	0.58	0.39	0.38	-
CI44	0.40	0.37	0.43	0.41	0.29	0.34

**Table 5:** The measure of the beta-diversity as indicated by the Jaccard distance.

Jaccard Distance	CI11	CI13	CI14	CI17	CI18	CI20
CI13	0.53	-	-	-	-	-
CI14	0.61	0.64	-	-	-	-
CI17	0.82	0.74	0.79	-	-	-
CI18	0.75	0.70	0.77	0.61	-	-
CI20	0.67	0.69	0.70	0.70	0.67	-
CI44	0.62	0.54	0.63	0.63	0.62	0.52

## Data Availability

The high-throughput amplicon sequencing reads of the 16S rRNA gene used in this study are available in the NCBI SRA database under accession numbers SRR13623317, SRR13623319, SRR13623320, SRR13623323, SRR13623324, SRR13623326 and SRR13623335.

## Supplementary Materials

The following are available online at https://www.mdpi.com/article/10.3390/microorganisms9091842/s1, Figure S1: Covering area of each sampling site and its corresponding features, Figure S2: Rarefaction curve of the seven samples, indicated by the number of ASVs depending on the size of the sequence sample, Figure S3: 16S rDNA V4-V5 phylogenetic tree showing the relationship between all selected reference alpha and beta-rhizobia strains and the 77 ASVs detected in savannah soils of Northern Côte d’Ivoire, Table S1: Physico-chemical properties of samples soils, Table S2: Levels of similarity between the V4-V5 sequences of the 77 ASVs of rhizobia and all 18 alphaproteobacterial and betaproteobacterial genera harboring rhizobia strains, Table S3: Major characteristics of the 77 ASVs of rhizobia detected in savannah soils of Northern Côte d’Ivoire, Table S4: ASVs and their corresponding md5_hash identities, Table S5: ASVs richness, cumulative relative abundance of sequences and prevalence of ASVs per soil, Table S6: Measure of the richness and the alpha diversity per savannah zone.
