# Supplementary material for: The Rhizobial Microbiome from the Tropical Savannah Zones in Northern Côte d’Ivoire
Source: Microorganisms. 2021 Aug 30;9(9):1842. doi: 10.3390/microorganisms9091842 (PMC8472840; doi:10.3390/microorganisms9091842)
Supplement: Supplementary file 1 [file microorganisms-09-01842-s001.zip › Gnangui et al, 2021_ Supplementary Figures & Tables_v1-correct.pdf]

Supplementary Materials

The rhizobial microbiome from the tropical savannah zones in Northern Côte d'Ivoire.

Sara Laetitia Elphège Gnangui, Romain Kouakou Fossou*, Anicet Ebou, Chiguié Estelle Raïssa Amon, Dominique Kadio Koua, Claude Ghislaine Zaka Kouadjo, Don A. Cowan and Adolphe Zézé

* Correspondence: romain.fossou@inphb.ci

Figure S1. Covering area of each sampling site and its corresponding features (not to scale).

Figure S2. Rarefaction curve of the seven samples, indicated by the number of ASVs depending on the size of the sequence sample.

Table S1. Physico-chemical properties of samples soils.

Table S5. ASVs richness, cumulative relative abundance of sequences and prevalence of ASVs per soil.

Table S6. Measure of the richness and the alpha diversity per savannah zone.

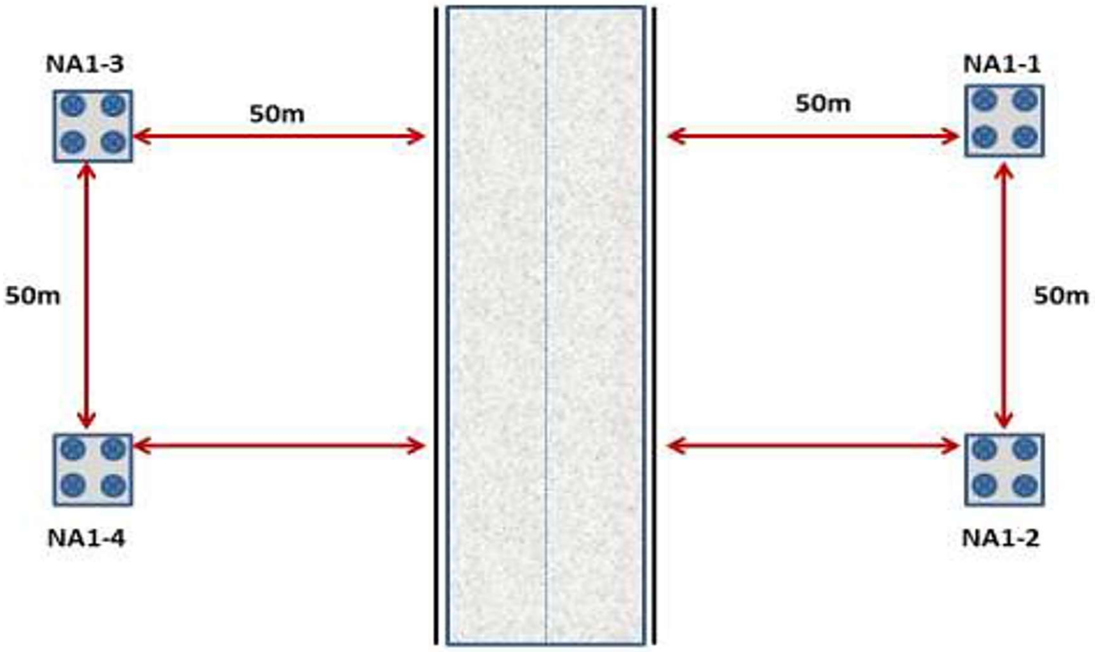

Figure S1. Covering area of each sampling site and its corresponding features (not to scale). Each sampling site was represented by an area of approximately. 100 m x 50 m with four independent sample locations (a virtual 1 m^2 quadrat) at the corners of the oblong. It is crossed in its length by a road of ca. 6 m wide. At each of the four independent sample locations, four topsoil cores (2 cm in diameter and 5 cm in depth) (pseudo-replicate samples) were collected, pooled together, and homogenized into a composite sample of approximately 25 g (replicate sample). Four independent replicate samples (4x25 g) obtained from four sample locations at each sampling site were kept in a labelled sterile plastic bag and formed an independent soil sample. A total of seven soil samples were collected following the same strategy for the seven sites studied in Northern Côte d'Ivoire.

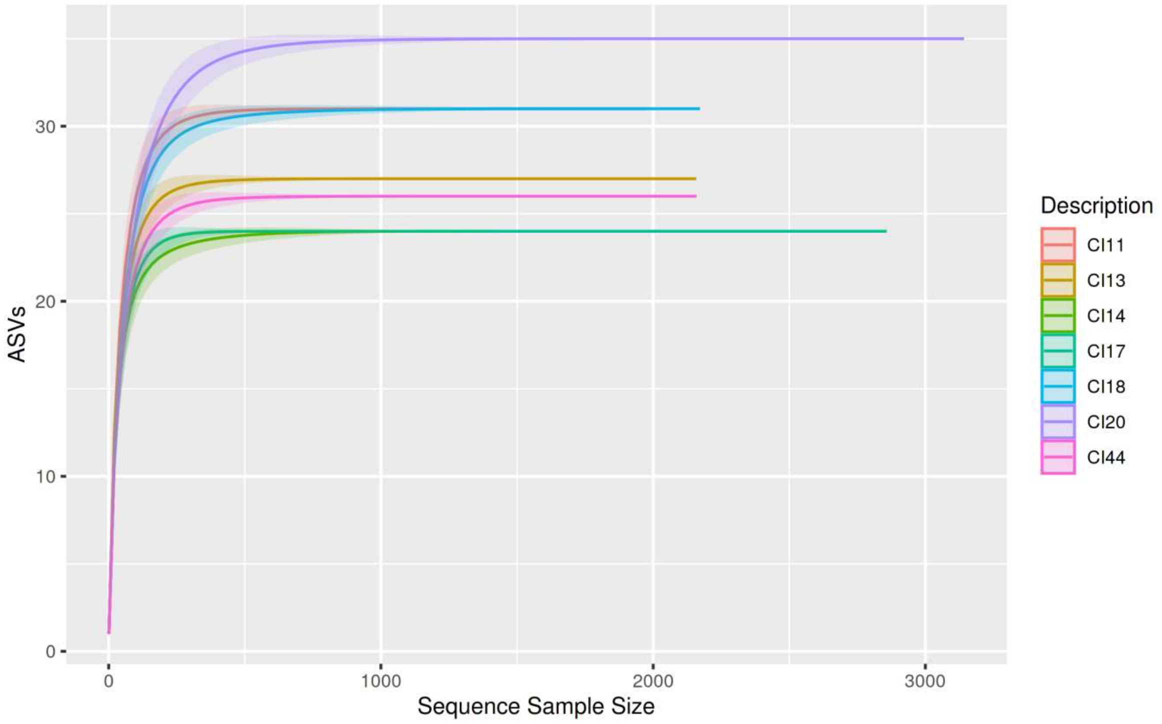

Figure S2. Rarefaction curve of the seven samples, indicated by the number of ASVs depending on the size of the sequence sample. Most rarefaction curves were able to reach the asymptote with a smaller number of sequences (<1000 sequences) than the subsampling size, suggesting that the sequencing effort and the subsampling size were appropriate.

Table S1. Physico-chemical properties of soil samples.

	Soil texture (%)			Mineral elements (mg/kg)						Other parameters			
**Soil ID**	**Clay**	**Sand**	**Silt**	**Al**	**Ca**	**Fe**	**K**	**Mg**	**Mn**	**Na**	**P**	**C (%)**	**pH (H_20)**
**CI11**	13	77	10	413.0	958.9	57.0	56.6	220.9	42.5	23.4	8.1	1.3	6.6
**CI13**	8	84	8	216.3	646.0	42.8	32.3	103.9	101.9	17.8	11.5	0.7	6.6
**CI14**	12	81	7	391.1	1230.2	66.4	59.5	175.3	63.2	22.1	14.6	1.4	6.4
**CI17**	12	78	10	461.0	254.8	93.3	24.5	88.9	183.1	20.3	5.3	0.6	6.0
**CI18**	12	80	7	495.7	819.7	48.4	84.2	155.1	38.5	21.1	16.4	1.2	5.8
**CI20**	14	72	14	414.7	359.7	83.5	30.3	104.7	37.2	19.8	7.3	0.7	6.0
**CI44**	12	85	3	241.9	338.0	43.4	22.8	85.4	49.2	12.5	22.7	0.6	5.7

Table S5. ASVs richness, cumulative relative abundance of sequences and prevalence of ASVs per soil. *Microvirga*, *Paraburkholderia* and *Bradyrhizobium* which are the most important genera in terms of ASVs richness, relative abundance and ubiquity are highlighted in bold.

				Prevalence of ASVs per soil						
**Genus (Final affiliation)**	**Number of ASVs per genus**	**Relative abundance of ASVs per genus**	**Cumulative relative abundance of sequences**	**Soil # CI11**	**# CI13**	**# CI14**	**# CI17**	**# CI18**	**# CI20**	**# CI44**
Microvirga	**24**	**31.17%**	**21.43%**	**12**	**13**	**9**	**4**	**9**	**5**	**5**
Parabukholderia	**11**	**14.29%**	**9.03%**	**3**	**2**	**1**	**5**	**5**	**4**	**5**
Bradyrhizobium	**9**	**11.69%**	**49.05%**	**5**	**4**	**4**	**5**	**5**	**8**	**5**
Ensifer	7	9.09%	1.23%	1	1	1	0	2	1	1
Cupriavidus	5	6.49%	5.14%	1	1	1	5	4	3	3
Trinickia	5	6.49%	2.57%	0	0	0	2	1	4	0
Methylobacterium	4	5.19%	1.72%	4	2	3	1	0	3	2
Mesorhizobium	3	3.90%	4.69%	2	2	1	1	2	3	3
Devosia	3	3.90%	2.68%	1	1	2	0	1	1	1
Rhizobium	3	3.90%	1.94%	1	1	1	1	2	2	1
Shinella	2	2.60%	0.31%	0	0	1	0	0	1	0
Neorhizobium	1	1.30%	0.19%	1	0	0	0	0	0	0
**Total**	**77**	**100%**	**100%**	**31**	**27**	**24**	**24**	**31**	**35**	**26**

Table S6. Measure of the richness and the alpha diversity per savannah zone. The richness is reported as the mean Chao1 index per soil and the alpha diversity is reported as the mean Shannon, Simpson and Fisher index per soil, with standard deviations shown in parentheses. All the richness and alpha diversity values were found to be statistically identical at the level =5 %.

Savannah zone	Chao1	Shannon	Simpson	Fisher
Sudanian savannah	27.33 (± 3.51)	2.91 ± (0.12)	0.92 ± (0.007)	4.55 (± 0.58)
Sub-sudanian savannah	29 (± 4.96)	2.77 (± 0.04)	0.89 (± 0.004)	4.59 (± 0.87)
